# EpCAM as multi-tumour target for near-infrared fluorescence guided surgery

**DOI:** 10.1186/s12885-016-2932-7

**Published:** 2016-11-14

**Authors:** P. B. A. A. van Driel, M. C. Boonstra, H. A. J. M. Prevoo, M. van de Giessen, T. J. A. Snoeks, Q. R. J. G. Tummers, S. Keereweer, R. A. Cordfunke, A. Fish, J. D. H. van Eendenburg, B. P. F. Lelieveldt, J. Dijkstra, C. J. H. van de Velde, P. J. K. Kuppen, A. L. Vahrmeijer, C. W. G. M. Löwik, C. F. M. Sier

**Affiliations:** 1 Department of Radiology, Division of Molecular Imaging, Leiden University Medical Centre, Leiden, Netherlands; 2 Percuros BV, Enschede, The Netherlands; 3 Department of Radiology and Division of Image Processing, Leiden University Medical Centre, Leiden, Netherlands; 4 Department of Surgery, Leiden University Medical Centre, Leiden, Netherlands; 5 Department of Otorhinolaryngology and Head and Neck Surgery, Erasmus Medical Centre, Rotterdam, Netherlands; 6 Department of Immunohematology and Blood Transfusion, Leiden University Medical Centre, Leiden, Netherlands; 7 Division of Biochemistry, Netherlands Cancer Institute, Amsterdam, Netherlands; 8 Department of Pathology, Leiden University Medical Centre, Leiden, Netherlands; 9 Antibodies for Research Applications BV, Gouda, The Netherlands

**Keywords:** Image-guided surgery, Near-infrared fluorescence, Optical imaging, Epithelial cell adhesion molecule, Imaging agent

## Abstract

**Background:**

Evaluation of resection margins during cancer surgery can be challenging, often resulting in incomplete tumour removal. Fluorescence-guided surgery (FGS) aims to aid the surgeon to visualize tumours and resection margins during surgery. FGS relies on a clinically applicable imaging system in combination with a specific tumour-targeting contrast agent. In this study EpCAM (epithelial cell adhesion molecule) is evaluated as target for FGS in combination with the novel Artemis imaging system.

**Methods:**

The NIR fluorophore IRDye800CW was conjugated to the well-established EpCAM specific monoclonal antibody 323/A3 and an isotype IgG1 as control. The anti-EpCAM/800CW conjugate was stable in serum and showed preserved binding capacity as evaluated on EpCAM positive and negative cell lines, using flow cytometry and cell-based plate assays. Four clinically relevant orthotopic tumour models, i.e. colorectal cancer, breast cancer, head and neck cancer, and peritonitis carcinomatosa, were used to evaluate the performance of the anti-EpCAM agent with the clinically validated Artemis imaging system. The Pearl Impulse small animal imaging system was used as reference. The specificity of the NIRF signal was confirmed using bioluminescence imaging and green-fluorescent protein.

**Results:**

All tumour types could clearly be delineated and resected 72 h after injection of the imaging agent. Using NIRF imaging millimetre sized tumour nodules were detected that were invisible for the naked eye. Fluorescence microscopy demonstrated the distribution and tumour specificity of the anti-EpCAM agent.

**Conclusions:**

This study shows the potential of an EpCAM specific NIR-fluorescent agent in combination with a clinically validated intraoperative imaging system to visualize various tumours during surgery.

## Background

Curative intended oncologic surgery aims to completely resect all malignant cells. Discriminating tumour from healthy tissue during surgery is therefore of paramount importance [1]. As a consequence, clinically complete resected tumours frequently turn out to be pathological incompletely removed [2–4]. Therefore, novel intra-operative imaging modalities are needed that aid the surgeon in recognizing tumour spread and provide guidance during tumour removal. Fluorescence-guided surgery (FGS) is a technique based on near-infrared (NIR) light, which has been widely investigated for sentinel lymph node procedures, anastomosis and during cholecystectomies [5–7]. The advantage of NIR fluorescent light is the relatively deep tissue penetration and the minimal tissue auto fluorescence at these wavelengths. The tissue depth at which a fluorophore can be detected is dependent on the fluorophore, the tissue optical properties and the sensitivity of the imaging device. Maximum tissue penetration detection has been estimated to be between 3 and 4 cm for intensified devices [8] and up to 2 cm for others [9]. Further, since NIR fluorescence light is invisible for the human eye, there is no alteration of the surgical field [10]. The major challenges for the routine introduction of FGS in the clinic comprise the availability of validated NIR fluorescence imaging systems in combination with a dedicated tumour-specific NIR fluorescence agent [11].

The first NIR imaging systems for clinical application proved the feasibility of the concept but were in fact more proto-types than standard clinical equipment. The next generation NIR imaging systems, as used in this study, are more versatile, smaller, cheaper, and more sensitive and should meet uniform standards warranting an exponential increase for clinical applications. Therefore, presently the biggest challenge for clinical introduction of FGS is the development of specific tumour targeting NIR fluorescent agents that comply with these second generation imagers. Various established membrane-bound tumour markers are under evaluation as targets for (NIR) fluorescence imaging in pre-clinical settings, such as EGFR, HER2/Neu, VEGF(R), folate receptor alpha, uPAR and various integrins [12–17]. Although these proteins have been successfully targeted in human tumours xenografted in animal models, none of them seems to be the universal target suited for the majority of tumour (types) in a clinical setting: These proteins are either present on the majority of tumours but only on a low percentage of tumour (stromal) cells, like VEGFR and α_v_β_3_ integrin, or they are abundantly present in only a limited percentage of tumour (types), like folate receptor, EGFR and HER2.

In this study we evaluate Epithelial Cell Adhesion Molecule (EpCAM) as target for FGS. EpCAM is a transmembrane glycoprotein involved in cell-cell interactions and cell-stroma adhesion [18]. EpCAM expression is restricted to epithelial cells and is highly up-regulated in virtually all epithelial carcinomas [19, 20]. EpCAM up-regulation is associated with cancer progression and EpCAM is found on circulating tumour cells and metastases. EpCAM overexpression in cancer cells was found to be 100- to 1000-fold higher compared to expression on normal breast cells resulting in 100,000 to 400,000 copies per cell [21]. Compared to an established tumour target like EGFR and Her2/Neu this number is only marginally less. But overexpression of EGFR and HER2/Neu in primary breast cancer is reported to be 0.8-14 and 15-20% respectively, whereas 40-98% of the primary breast tumours show enhanced EpCAM levels [20, 22]. For other epithelial cancers these figures are similar or favour the use of EpCAM as imaging target even more [20, 23–25]. These findings have led to the development of many EpCAM specific antibodies, from which some have been evaluated in phase I, II and III immunotherapeutic trials in various cancer types, such as ovarian, gastric and head-and-neck cancer [26, 27]. The results of these studies did not meet the high expectations for the therapeutic purpose and most of these investigations were aborted. Mild adverse effects like nausea, vomiting and elevation of pancreatic enzymes have been reported for therapeutic use, but are not expected in single dose imaging studies. The potential of EpCAM targeting has recently been re-discovered, as tumour specific imaging was demonstrated for SPECT imaging. A monoclonal antibody with medium high affinity for EpCAM, has been labelled with various radionuclides and has been extensively evaluated in several xenografted tumour models in mice [28, 29].

In the current study we conjugated the same monoclonal antibody 323/A3 to the clinically relevant NIR fluorescent dye IRDye800CW and evaluated this conjugate in tumour models of clinically relevant orthotopic colorectal, breast and head-and-neck cancer models. To simulate clinical conditions we evaluated the performance of the probe with the recently introduced commercially available Artemis imaging system [30] in comparison with the Pearl Impulse small animal imaging system, a standard apparatus for use in pre-clinical settings.

## Methods

### Cell lines

For colorectal, breast and head-and-neck cancer, we selected two cell lines with different tumour characteristics. Luciferase transfected cells were used to follow orthotopic tumour growth by bioluminescence imaging (BLI). MCF-7 and OSC-19 cells were transfected with Luciferase 2 and green fluorescent protein as described previously [31]. All cell lines were grown in a humidified incubator at 37 °C and 5% CO_2_. Cells were cultured for not more than 10 passages and regularly checked for *Mycoplasma* infection by PCR.

### EpCAM expression

EpCAM expression of HT29(−/+)luc2, COLO320, OSC-19-luc2-cGFP, FaDu-luc2, MCF-7-luc2-cGFP and MDA-MB-231 cells was evaluated by flow cytometry. Cells were cultured until 90% confluence and detached with trypsin. Viability of the cells was evaluated using trypan blue. After adjusting the number of cells to 0.5 × 10^6^ per tube in ice cold phosphate-buffered saline (PBS), they were incubated with 0.4 μg/ml 323/A3 anti-EpCAM antibody or isotype control MOPC21 for 30 min on ice. Then cells were washed three times in ice cold PBS and incubated with a goat anti-mouse IgG1-AF488 secondary antibody (Invitrogen, 2.5 μg/ml). The cells were washed three times in ice cold PBS and resuspended in 400 μL PBS containing propidium iodide to exclude dead cells from the analysis. Flow cytometry was performed using the LSRII (BD Biosciences). The experiments were performed in duplicate and EpCAM expression was estimated as the geometric mean of fluorescence intensity measured in 10,000 viable cells. For quantitative determination of EpCAM numbers per cell type the Qifikit (Dako) was used.

### Antibodies and conjugation to IRDye 800CW

EpCAM specific monoclonal chimeric antibody 323/A3 and the IgG_1k_ isotype control monoclonal antibody MOPC21 (BioXcell, West Lebanon, USA) were used [32]. Antibody 323/A3 has a medium high affinity (K_α_ = 2 × 10^9^ M^−1^) for EpCAM and is directed against the EGF-like domain I epitope on the extracellular domain of the EpCAM molecule, whereas MOPC21 has an unknown specificity after testing on human and rodent tissues [33–35]. Both antibodies were covalently conjugated to NIR fluorochrome IRDye 800CW (LI-COR, Lincoln, NE, USA). λex = 773 nm, λem = 792 nm) using N-hydroxysuccinimide ester chemistry as indicated by the manufacturer. Removal of unconjugated fluorophore was accomplished by using two Zeba Spin Desalting columns (Thermo Fisher Scientific, Perbio Science Nederland B.B., Etten-Leur, The Netherlands) per protein in two sequential steps. For comparison experiments, the two conjugates i.e. the EpCAM specific (323/A3-800CW) and control (MOPC21-800CW) were complemented by the chemically inactive carboxylate version of IRDye 800CW, representing the fluorescent label without antibody control.

### Serum stability

The stability of 323/A3-800CW in human serum was evaluated using HPLC (Biosep-SEC-s2000, Phenomenex, USA). Serum and sodium azide dilution were filtrated through a 0.22 μm filter in a 15 ml tube. A 24-wells plate (Greiner Bio-one, Germany) was prepared with 0.02% sodium azide and serum/probe in a ratio of 1:1 and PBS as control and incubated at 37 °C under 5% CO_2_. At 4, 24, 48 and 96 h 20 μl of sample, diluted in 40 μL PBS was evaluated using HPLC in PBS at a flow rate of 0,5 ml/min for 60 min, detected at 2 channels, 280 and 780 nm.

### Cell binding study

A cell binding assay was performed to confirm the EpCAM specificity of 323/A3-800CW. HT29-luc2 (40,000 cells), COLO320 (40,000 cells), OSC-19-luc2-cGFP (25,000 cells), FaDu-luc2 (35,000 cells), MCF-7-luc2-cGFP (40,000 cells) and MDA-MB-231 cells (40,000 cells) cells were seeded in a black 96-well plate (Greiner Bio-one, Germany). At ±90% confluence the cells were washed twice with PBS. 323/A3-800CW and MOPC21-800CW were added in a concentration range of 0–8 μg/ml and incubated for 1 h at 37 °C. After incubation, the cells were washed twice with culture medium without supplements. Bound antibody was imaged with an Odyssey scanner (LI-COR), scanning at the 800 nm channel. To correct the fluorescence signal for the number of tumour cells per well a cell nucleus staining was performed: The cells were fixed/permeabilized with acetone/methanol for 10 min, washed with PBS, and incubated with TO-PRO-3 (Invitrogen) at 1:1000 for 5 min at room temperature. After washing twice with PBS, the plate was imaged with the Odyssey scanner at the 700 nm channel to detect TO-PRO-3 fluorescence. The ratio of the 800 and 700 nm fluorescence was plotted. The experiments were performed in triplicate.

### Animal models

Nude Balb/c female mice (Charles River laboratories, l’Arbresle, France), aged 4–6 weeks, were housed in individually ventilated cages and provided with food and sterilized water *ad libitum*. Their general health state was monitored by weight measurements throughout the experiments. Tumour growth was monitored longitudinally by visual inspection of the tumours, caliper measurements and/or by bioluminescence imaging. Bioluminescence imaging was performed by intraperitoneal injecting of 150 mg/kg of D-luciferin solution (SynChem, Inc, Elk Grove Village, IL) in PBS, in a total volume of 50 μL. After 10 min, mice were imaged with the IVIS Spectrum imaging system (PerkinElmer, Waltham, MA, USA). Imaging procedures were performed under isoflurane gas anaesthesia

- Colon cancer models
  To induce colon tumours, mice were subcutaneously injected at four sites with 5 × 10^5^ HT29 cells in 40 μL RPMI1640 medium. Tumour growth was followed by calliper measurements and after 10 days, when the tumours reached a volume of approximately 75 mm^3^, imaging experiments started. Orthotopic HT29-luc2 tumours were induced as described previously [36].
  To induce orthotopic breast tumours, 2.5 × 10^5^ MCF-7-luc2-cGFP cells were inoculated in two contralateral mammary fat pads. Oestrogen pellets (17β-oestradiol, 0.36 mg/pellet, 60 day release) were implanted subcutaneously. Tumour growth was followed by visual inspection and bioluminescence measurements as described above.
- Breast carcinoma model
  Orthotopic tongue tumours were induced in the tip of the tongue through a submucosal injection of 4 × 10^4^ OSC-19-luc2-cGFP cells. When tumours were visible and bioluminescence signal ranged between 5 × 10^9^ and 1 × 10^10^ relative light units (RLU) imaging experiments started.
- Peritonitis carcinomatosa
  Multiple small MCF-luc2-cGFP tumours were induced in the peritoneum by intraperitoneal injection of 2.5 × 10^5^ MCF-luc2-cGFP cells. Tumour growth was followed twice a week by bioluminescence as described above. Imaging experiments initiated when multiple tumour nodules were formed of various sizes.

### NIR fluorescence imaging systems

Real-time NIR fluorescence imaging and operative resection of the tumours was performed using the next generation Artemis imaging system (Quest Medical Imaging, Middenmeer, the Netherlands). An earlier iteration of this system was extensively validated [30]. This system has a freely moveable handheld camera for simultaneous acquisition of visible light and NIR fluorescence. Since the system has been improved such that the camera can be fixed in a stable position with an arm, while both camera and arm are covered with a sterile drape. The illumination efficiency and homogeneity has been improved with a ring containing eight hemispheric illumination lenses centred around a wide field imaging lens for open surgery. Illumination is provided by four visible light sources with peaks centred in the blue, cyan, green and red and a NIR laser with a peak at 785 nm for fluorescence excitation. Reflected excitation light is blocked by a 750–800 nm notch filter. Captured visible and NIR light is split using a prism containing a dichroic coating (<785 mm). Visible light additionally passes through a low-pass filter (<640 nm) and the NIR emission light is filtered with a high pass filter (>808 nm). Exposure times and sensor gains are separately adjustable for both imaging channels. The visible light channel, the NIR fluorescence channel and an adjustable overlay of both channels are simultaneously presented during the procedures.

Next to the Artemis imaging system, the Pearl Impulse small animal imaging system (LI-COR) was used as a preclinical reference to visualize tumours and calculate the tumour-to-background ratios (TBRs). Data from the Artemis and Pearl imaging systems were analysed using imageJ (W. Rasband, Bethesda, Maryland) and the Pearl Cam Software, respectively.

### NIR fluorescence measurements

Subcutaneous HT29 colon tumours were used to confirm in vivo EpCAM specificity of 323/A3-800CW and to measure fluorescence over time. When the subcutaneous HT29 colon tumours were 36 ± 6 mm^2^, 1 nmol (≈150 μg) of 323/A3-800CW (*n* = 3), 1 nmol (≈150 μg) MOPC-800CW (*n* = 3) or 1 nmol (≈1.1 μg) of 800CW carboxylate was injected intravenously. NIR fluorescence signals were measured at 0, 4, 24, 48, 72 and 96 h after injection using the PEARL small animal imaging system and the intraoperative Artemis imaging system after which TBRs were calculated.

After in vivo confirmation of the EpCAM specificity and establishment of the optimal time frame for imaging with the subcutaneous model, the clinically more relevant orthotopic MCF-7-luc2-cGFP breast, OSC-19-luc2-cGFP tongue and HT29-luc2 colon tumours were evaluated. Hence, 1 nmol of 323/A3-800CW (*n* = 3) or 1 nmol MOPC21-800CW (*n* = 3) was intravenously injected in each group. Fluorescence imaging of mice bearing orthotopic tumours was performed 72 h after administration for optimal TBR as determined in the subcutaneous HT29 colon carcinoma model. TBRs of orthotopic tumours were measured and tumours were resected under NIR fluorescence guidance using the Artemis imaging system. *Ex vivo*, fluorescent measurements of resected tissue were performed on a back table. Tumours were sliced and fluorescence measurements were performed on the sections to evaluate the distribution of the probe. Resected tumours from the head-and-neck cancer and breast cancer models were assessed by BLI imaging and GFP fluorescence imaging (OSC-19-luc2-cGFP and MCF-7-luc2-cGFP, IVIS spectrum).

The MCF-luc2-cGFP peritonitis carcinomatosa tumour model was used to determine the minimal tumour sizes that could be detected by intra-operative fluorescence imaging using the EpCAM specific antibody 323/A3-800CW in combination with 2 imaging systems. Because an enhanced permeability and retention (EPR) effect in these micrometases is not expected as indicated recently by Hall et al. [34] no MOPC21-800CW control was used in this model. Mice were anesthetized 72 h after intravenous injection of 323/A3-800CW (1 nmol, *n* = 3), as described above and fluorescence imaging using the Pearl and Artemis imaging system was performed. A midline abdominal incision was made and the abdominal skin was removed. Fluorescence imaging of the mice with both imaging systems was performed followed by resection of the peritoneum and again fluorescence images were taken. Fluorescence imaging of the abdominal area was performed to search for residual intraperitoneal tumour nodules. Presence of tumour nodules and tumour specific NIR fluorescence was confirmed by bioluminescence imaging and GFP fluorescence imaging, as described before. Receiver Operator Curve (ROC) analysis was performed for the detection of micrometastases in the peritoneum. The overlay of the BLI and GFP signals was used as the ground truth for tumour metastases and the ascending TBRs as positive cut off criteria. The area under the curve (AUC), including the sensitivity and specificity rates at the optimal TBR cut off were computed. The signals of NIR fluorescence and the overlay with BLI and GFP was confirmed against pathology in the primary tumor but not in the micrometastases.

Multiple regions of interest were drawn in the tumour and in adjacent normal tissue and divided by each other to calculate TBRs. TBRs of subcutaneous colon and orthotopic breast tumours were calculated with skin overlying the tumour and adjacent normal tissue. Tongue tumours were imaged through an epithelial cell layer covering the tumour and normal tissue. For colon tumours the peritoneum was opened.

### In vivo competition study

Three of the six nude Balb/C mice with bilateral orthotopic MCF-7-luc2-cGFP breast tumours were pre-injected with unconjugated 323/A3 antibody (1 mg, intraperitoneal, 100 μL). After 48 h, all six mice were intravenously injected with 1 nmol 323/A3-800CW. Then, 72 h after injection of 323/A3-800CW fluorescence imaging of all mice was performed using a Pearl imaging system. Mice were sacrificed and tumours were collected. Quantification of fluorescence was done as described before [37]. In brief, tumours were resected and lysed with a TissueLyser II system (Qiagen, Venlo, The Netherlands) using pre-cooled Eppendorf tube holders, 5-mm stainless steel beads, and RIPA buffer supplemented with a complete EDTA-free mini tablet protease inhibitor cocktail. Homogenates were serially diluted in 96-well plates, in parallel with a probe dilution. The fluorescence intensity of both series was detected at 800 nm using the Odyssey scanner. The concentration of probe in the homogenates was extrapolated from the calibration curves and the concentration values were used to calculate the injected dose per gram of tissue (% ID/g) with standard error of the mean (SEM) indicated per group, based on the volume of the homogenate and the weight of the tumours.

### Histology and NIR fluorescence microscopy

After *ex vivo* fluorescence measurements, tumours were snap frozen in isopentane and kept at −80 °C. Tissues were sectioned at 10 μm and fluorescence imaging was performed using the Odyssey imager. The presence of OSC-19-luc2-cGFP and MCF-7-luc2-cGFP tumour cells was confirmed by fluorescence microscopy (Nikon Eclipse e800). All histologic sections were stained with standard haematoxylin-eosin stain (HE) after acetone fixation. To confirm the presence of HT29 and HT29-luc2 cells, sections were stained with an anti-human wide-spectrum cytokeratin antibody (Abcam inc., Cambridge, MA, USA). Primary antibodies or controls were incubated for 60 min at room temperature. All slides were three times washed with PBS and incubated with Envision anti-rabbit (DAKO) for 30 min at room temperature. Subsequently, the slides were washed with PBS and staining was visualized by using 3,3-diaminobenzidine. Sections were counterstained with haematoxylin, dehydrated and mounted with pertex. Frozen OSC-19-luc2-cGFP and MCF-7-luc2-cGFP tumours were stained with an anti-cGFP staining (Evrogen, Moscow, Russia). Sections stained with anti-cGFP were fixated with 4% formalin for 10 min. After washing with PBS, cells were treated with 0,1% saponin/PBS for 10 min and incubated with the anti-GFP antibody, diluted in 0,1% saponin/PBS for 60 min at room temperature. Adjacent sections were fixated with aceton for 10 min followed by three washes with PBS and stained for cytokeratin as described before.

### Statistical analysis

For statistical analysis, SPSS statistical software package (version 20.0 for Windows, IBM SPSS Inc, Chicago, USA) was used. TBRs were calculated by dividing the fluorescent signal of the tumour by fluorescent signal of surrounding healthy tissue. TBRs are reported in mean and standard deviation. A two-way repeated measurement ANOVA was used to assess the relation between TBRs in the dose groups and time points. Furthermore a paired Student’s *t*-test was used to calculate the overall difference between the EpCAM specific and control groups. The two-way repeated measurement ANOVA was corrected using the Bonferroni correction. A *P*-value equal or lower than 0.05 was considered significant.

## Results

### EpCAM expression on human cancer cell lines

**Fig. 1:**
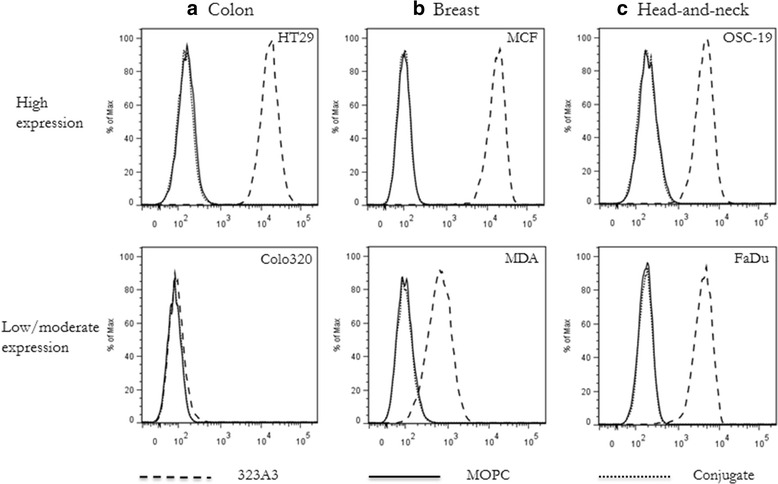
EpCAM expression: EpCAM overexpression was present in all cell lines except in COLO320 cells. Expression of EpCAM was analyzed in human colon (**a** HT29-luc2, COLO320), breast (**b** MCF-7, MDA-MB231) and head & neck (**c** OSC-19, FaDu-luc2) cancer. Cells were incubated with an anti-EpCAM antibody (323/A3) or an isotype normal mouse IgG (MOPC21). After washing, cells were incubated with fluorescein isothiocyanate (FITC)-conjugated anti-mouse IgG antibody. The anti-mouse antibody was solely used as a control (conjugate)

The colon adenocarcinoma cell line HT29 showed intermediate high expression of EpCAM while COLO320 hardly had any EpCAM expression (200,000 versus <1000 EpCAM/cell), see Fig. 1a). For MCF-7 breast cancer cells similar EpCAM over-expression was observed as for HT29, whereas considerably less expression was seen in MDA-MB-231 cells, as described previously (Fig. 1b). For head-and-neck cancer cells, the human hypopharyngeal squamous cell carcinoma cell line FaDu-luc2 and the oral squamous cell carcinoma cell line OSC-19 showed similar intermediate EpCAM expression (Fig. 1c). For all cancer cell lines the control antibody MOPC21 showed no significant signal. EpCAM expressing cell-lines MCF-7 and OSC-19 were transfected with GFP and luciferase and were, next to HT29-luc2 used for further in vivo experiments.

### Conjugation, characterization and serum stability of 323/A3-800CW

**Fig. 2:**
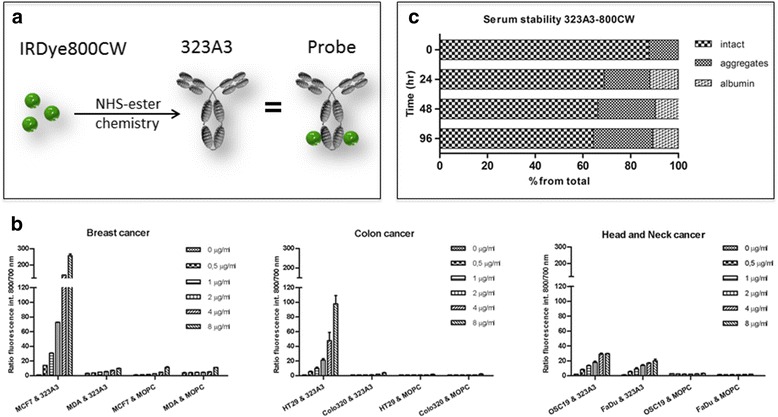
Conjugation, serum stability and EpCAM specificity: Conjugation of IRDye 800CW to 323/A3 and MOPC21 was done through NHS ester conjugation. A mean labeling ratio of 2.6 ± 0.98 and 2.8 ± 0.99 was obtained for 323/A3 and MOPC21 respectively **a**. EpCAM specificity of 323/A3 was confirmed after conjugation of IRDye 800CW on various cell types, corrected for cell number using the 800/700 nm ratio **b**. Experiments were done in human colon (HT29-luc2, COLO320), breast (MCF-7-luc2-cGFP, MDA-MB231) and head & neck (OSC-19-luc2-cGFP, FaDu-luc2) cancer. Cells were incubated with different concentrations of 323/A3-800CW or an isotype normal mouse IgG (MOPC21) conjugated to 800CW. To correct for the number of cells, the ratio of NIR fluorescence and the fluorescence intensity of TO-PRO-3 was plotted. 323/A3-800CW showed to be stable in human serum with more than 60% of the conjugate still free to bind EpCAM after 96 h with the remaining 40% aggregated or bound to albumin **c**

Both the EpCAM specific (323/A3) and the control antibody (MOPC21) were labelled with IRDye 800CW (Fig. 2a) in comparable mean dye:antibody ratios of respectively 2.6 ± 0.9 and 2.8 ± 0.9, as determined spectrophotometrically, according to the protocol of the manufacturer of the dye. Plate assays containing both high and low EpCAM expressing cell lines were utilized to confirm EpCAM specificity of 323/A3-800CW after conjugation. A significant (*p* < 0.01) difference was observed between 323/A3-800CW and MOPC21-800CW signals on MCF-7, HT29, OSC-19 and FaDu cells, but not on the low EpCAM expressing COLO320 and MB-MDA-231 cells (Fig. 2b). The fluorescence intensity of MOPC21-800CW showed a slight concentration dependent increase as a result of non-specific binding. The results are in accordance to the EpCAM expression found using flow cytometry results of the antibodies without 800CW label, as shown in Fig. 1. The increase in concentration resulted in a plateau in fluorescence intensity in MDA-MB-231, OSC-19 and FaDu cells. A further increase in concentration did not increase signal intensity. This phenomenon was not seen in MCF-7 and HT29 cells, possibly due to the high EpCAM expression. The EpCAM specific conjugate showed to be stable in human serum with more than 60% of the conjugate still free to bind EpCAM after 96 h with the remaining 40% aggregated or bound to albumin (Fig. 2c).

### Intra-operative NIR fluorescence tumour delineation and resection

**Fig. 3:**
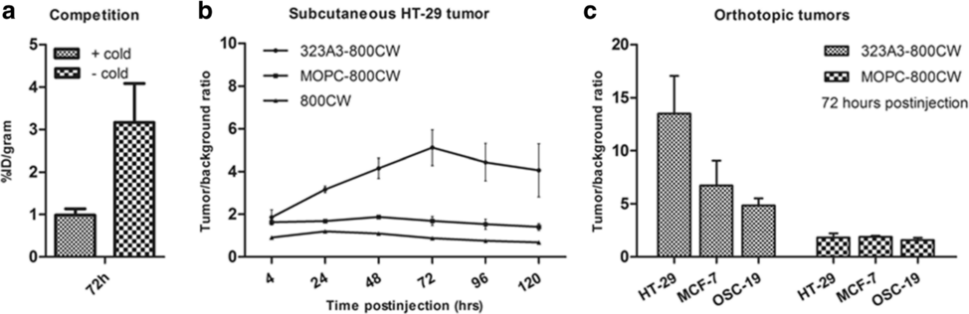
EpCAM specificity and intra-operative tumor-to-background ratios (TBR’s): Competition with 1 mg of cold 323/A3 antibody significantly decreased the percentage of injected dose 323/A3-800CW per gram tumor (%ID/g) indicating in vivo tumor specificity **a**. After development of subcutaneous HT29-luc2 tumors, 1 nmol (150ug) of 323/A3-800CW, MOPC21-800CW and IRDye 800CW was injected intravenously. TBR’s were calculated from 4 h to 120 h after injection **b**. Subsequently, 1 nmol (150ug) 323/A3-800CW and MOPC21-800CW was intravenously injected in mice bearing orthotopic colon (HT29-luc2), breast (MCF-7-luc2-cGFP) and tongue (OSC-19-luc2-cGFP) tumors **c**. TBR’s were calculated 72 h post injection. TBR’s of EpCAM specific antibody 323/A3-800CW in colon, breast and head & neck cancer are significantly higher compared to those of the control antibody MOPC21-800CW

In vivo specificity of 323/A3-800CW was shown by competition with unconjugated antibody. Pre-injection of 1 mg unconjugated 323/A3, resulted in a decrease of the percentage of injected dose per gram of tumour from 3.2 ± 0.9% ID/g to 1.0 ± 0.1% ID/g (*p* < 0.01, *n* = 3 in both groups), see Fig. 3a. The HT29 subcutaneous tumour model was used to assess the feasibility of intraoperative fluorescence delineation using 1 nmol (150ug) intravenous 323/A3-800CW combined with the Artemis imaging system. Tumours could be clearly delineated from 4 h post injection and an increase in TBR was seen with highest values at 72 h post-injection (5.2 ± 0.7, Fig. 3b). After 72 h the TBR slightly decreased. Significant differences in TBR (*p* < 0.01) between EpCAM specific (323/A3-800CW) and non-specific control (MOPC21-800CW) could be observed from 24 h post injection (TBR 3.2 ± 0.1 and 1.7 ± 0.2 respectively). MOPC21-800CW and IRDye 800CW showed a significantly lower TBR at all time points (*p* < 0.01).

**Fig. 4:**
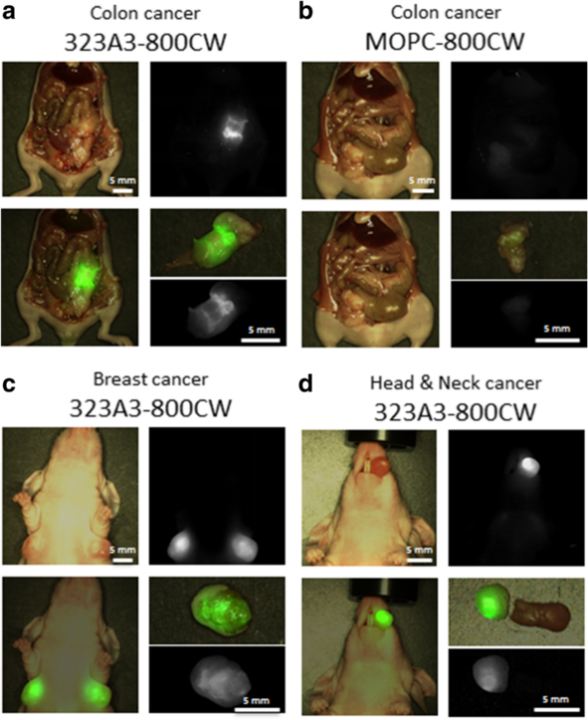
Intra-operative NIR fluorescence delineation of colon, breast and Head & Neck cancer: Colon **a**, **b** breast **c** and head & neck tumors **d** could clearly be visualized during operation using EpCAM specific 323/A3-800CW (TBR colon 13.5, breast 6.7 and Head-and-neck 4.9) and the Artemis imaging system. 323/A3-800CW (A, C, D, 1 nmol) and the non-specific antibody MOPC21-800CW (B, 1 nmol) were intravenously injected. After an incubation of 72 h NIR fluorescence imaging was performed. Depicted images are: bright light, fluorescence, overlay (fluorescence and bright light), *ex vivo* fluorescence and *ex vivo* overlay. Scale bars are 5 mm. Artemis fluorescence intensities were intra-operatively optimized for optimal contrast. Intensities can only be compared within images

Based on the results from the subcutaneous tumour model an optimal incubation time of 72 h was used to evaluate NIR fluorescence in clinically more realistic orthotopic mouse models for colon, breast and head-and-neck cancer. After exploration of the tumour, we assessed the feasibility of fluorescence-guided resection and chose suitable resection margins with the real-time NIR fluorescence feedback of 323/A3-800CW and the Artemis imaging system. All tumours were radically resected as confirmed by BLI evaluation. Significant differences (*p* < 0.01) in TBRs were observed between the orthotopic colon (HT29) mice injected with 323/A3-800CW (TBR 13.5 ± 3.6) and MOPC21-800CW (TBR 1.8 ± 0.4) (Fig. 3c). For the breast (MCF-7) and head-and-neck (OSC-19) cancer models, 323/A3-800CW showed TBRs of 6.7 ± 1.9 and 4.9 ± 0.7 respectively (Fig. 3c). Significantly lower TBR values of 1.9 ± 0.1 and 1.6 ± 0.2 were observed for MOPC21-800CW in breast and head-and-neck cancer respectively (*p* < 0.01). Examples of in vivo and *ex vivo* images that were acquired with the Artemis imaging system after injection of 323/A3-800CW or MOPC21-800CW are shown for the orthotopic colon (Fig. 4a and b), breast (Fig. 4c) and head-and-neck cancer model (Fig. 4d). Camera exposure time and gain were intra-operatively adjusted to obtain optimal image contrast. The most important factors influencing measured fluorescent intensities were sample fluorescence intensity, camera settings and the coupled distance to sample of both illumination source and camera. The Artemis camera is not calibrated for absolute intensity measurements (e.g. in lumen), hampering absolute intensity comparisons between images.

### Detection of small tumour sizes

**Fig. 5:**
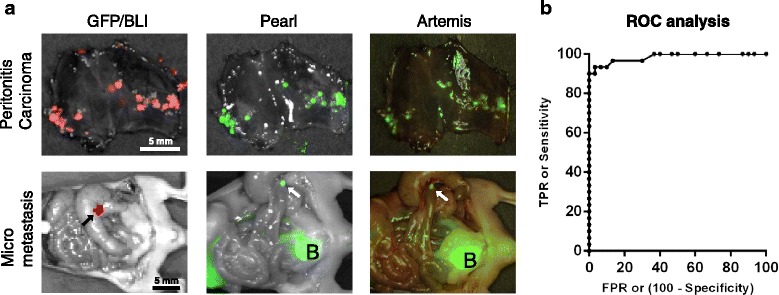
Visualization of submillimeter sized intra-peritoneal metastases using EpCAM specific 323/A3-800CW in combination with the Artemis system: **a** MCF-7-luc2-cGFP cells were injected intraperitoneal in mice. After the development of multiple tumors, 1 nmol (150ug) 323/A3-800CW was injected intravenously. NIR fluorescence imaging was performed after 72 h of incubation using the Pearl and Artemis system. Confirmation of tumor tissue was performed by Green Fluorescent Protein (GFP) fluorescence imaging or Bioluminescence Imaging (BLI) using the IVIS spectrum. Micrometastases (arrow) of the colon could be discovered by NIR fluorescence imaging after resection of the peritoneum. **b** ROC curve analysis of TBR of fluorescent spots in the peritoneum. True positive rate was plotted versus false-positive rate using ascending positive cutoff values. B = bladder. Scale bars are 5 mm. Artemis fluorescence intensities were intra-operatively optimized for optimal contrast. Intensities can only be compared within images

Four weeks after the intra-peritoneal injection of MCF-7-luc2-cGFP cells multiple tumours were observed by BLI imaging. Millimetre size tumour nodules located on the peritoneum, which were not visual by the naked eye, could be observed by NIR fluorescence imaging after injection of 1 nmol (150ug) 323/A3-800CW, using both the Pearl and Artemis imaging system (Fig. 5). After the peritoneum was resected, BLI confirmed that intra-peritoneal metastases could be detected by NIR fluorescence imaging (Fig. 5a). As in Fig. 4, camera exposure time and gain were adjusted to obtain optimal contrast. ROC curve analysis showed an accuracy of 98% (area under the curve, *p* < 0.0001) for detecting micro metastases with a sensitivity of 93% and specificity of 92% at the curve’s optimal TBR cut off value (Fig. 5). A cut off value obtaining the highest sensitivity of 100% correlates with a specificity of 73%. A specificity of 100% on the other hand correlates with a sensitivity of 84%.

### Histology and Immunohistochemistry

**Fig. 6:**
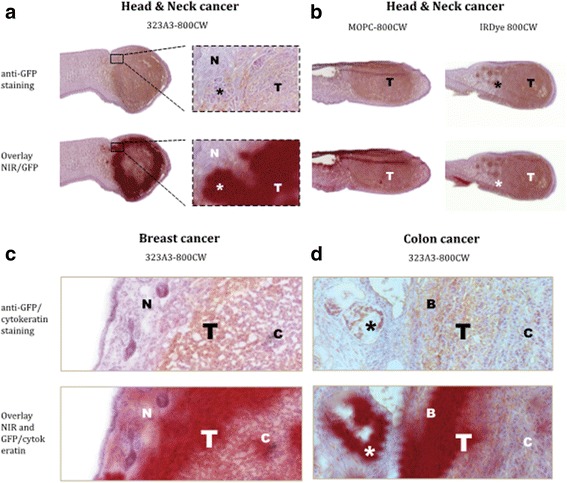
Histology: 323/A3-800CW is specifically located in head-and-neck **a**, **b**, breast **c**, and colon **d** tumors. Tumor tissue was sectioned 72 h after injection of 323/A3-800CW, MOPC21-800CW or IRDye 800CW. Shown are anti-GFP (brown, head-and-neck cancer, breast cancer) or cytokeratin (brown, colon cancer) immunohistochemistry stainings and overlays of NIR fluorescence (red) and anti-GFP/cytokeratin stainings. In all tissue a clear overlap is seen between NIR fluorescence from 323/A3-800CW and anti-GFP immunohistochemistry staining (indicating tumor). NIR fluorescence is mainly located in the border of tumors and even small tumor islands (*) are NIR fluorescent. A low, non specific fluorescence signal was observed in sections with MOPC21-800CW and IRDye 800CW (B). T = tumor; N = normal tissue; C = center tumor; B = tumor border. A and B 1× zoom; C and D 10× zoom

Orthotopic tumour tissue was harvested 72 h after injection of 323/A3-800CW, MOPC21-800CW and IRDye 800CW. In sections of cGFP transfected breast and tongue tumours, the presence of tumour tissue was confirmed by anti-cGFP immunohistochemical staining (brown colour, Fig. 6c and d). Fig. 6a clearly illustrates NIR fluorescence (indicated in red) intensity and localization of 323/A3-800CW, MOPC-800CW and IRDye 800CW in tissue sections of tongue tumours, surrounded by normal tissue of the tongue. Fluorescence of 323/A3-800CW co-localized with the outer part of the tumour bulk and with small peninsulas of tumour cells (Fig. 6a). Fluorescence intensity of MOPC21-800CW and IRDye 800CW was hardly visible and did not show association with tumour tissue (Fig. 6b). In both, breast and colon cancer tissue sections NIR fluorescence appeared in the outer rim of tumour tissue. (Fig. 6c and d). In colon cancer clear co-localization of small tumour islands and NIR fluorescence (indicated in red) was observed with 323/A3-800CW (Fig. 6d). Cytokeratin staining (brown) was performed to confirm the presence of colonic tumour cells in sections of orthotopic colon tumours (Fig. 6d).

## Discussion

In this study we investigated an EpCAM-specific NIR-fluorescent agent using a state-of-the-art next generation clinical NIR imaging system. The recent technical developments stimulate the use of advanced but affordable imaging systems in the clinic. The Artemis imaging system simultaneously acquires NIR-fluorescent and visible light, generating a merged image in real-time. Systems like this will boost the demand for specific tumour probes dramatically.

The EpCAM directed antibody in this study has been extensively evaluated for imaging of various tumour models using radionuclides Technetium, Zirconium, Iodium and Rhenium [28, 29, 38]. Typically, in these experiments 15–20 μg of conjugate was used per mouse to obtain clear tumour signals in subcutaneous breast, colon and ovarian tumours. Using the same antibody but conjugated to a NIR dye and in combination with a state of the art NIR-fluorescence imager, we showed that 1 nmol (=150 μg) clearly identified breast, colon and head-and-neck cancers, as well as micro-metastases in human orthotopic xenograft mouse models. The probe/system combination allowed an accurate demarcation of the tumours, the recognition of tumour margins, examination of malignant spread, and identification of micrometre sized metastasis or remnant disease.

A probe against a ‘universal’ target facilitates clinical usability, and prevents time consuming and costly development of multiple agents. Although EpCAM is considered to be a potential target for epithelial derived cancers, not all epithelial cancers over-express EpCAM enough to outflank adjacent normal tissue. But on the other hand not all tumours of mesenchymal origin are EpCAM negative. Over-expression of EpCAM was recently observed in all osteosarcomas, half of the angiosarcomas and 62.5% of the leiomyosarcomas, indicating the broad spectrum of tumours that might be targeted [39].

In breast cancer, 20% of tumours that seem radically resected during surgery turn out to be irradically removed in histologic analyses. Consequently, FGS in breast cancer could enhance the number of radical resections without increasing margins, thus improving patient’s prognoses and cosmetic outcomes after lumpectomies and oncoplastic surgery [3, 40]. As pointed out earlier, in copies per cell EpCAM can probably not compete with Her2/Neu in the subpopulation of Her2 positive breast cancers, but in percentage of positive breast tumours EpCAM is clearly the more prevalent target. For head-and-neck cancer a relatively low percentage of tumours is irradically resected (16%). Due to the many vital structures in the surrounding tissue, resection margins should be small and FGS targeting would be of great clinical benefit. EpCAM overexpression is seen in 62.5% of tongue tumours [23, 41]. In colorectal cancers 80-100% overexpression of EpCAM is found. Although irradical resections occur, the main problem is damage to vital structures in the lower abdomen. Ureteral injury is a rare but serious complication of lower abdominal surgery, with a reported incidence varying from 0.7 up to 10% [42]. EpCAM-based FGS of colorectal tumours in combination with FGS of the ureters could enhance the number of radical resections while preserving vital structures.

In our study, we performed a single injection of 1 nmol (150ug) of 323/A3-800CW to visualize tumours. This is the equivalent of 0.5 mg/kg for humans when converted using the body-surface-area method [43]. Using this dose, subcutaneously located colon tumours were clearly recognized from 4 h after injection with optimal TBRs at 72 h, showing the feasibility of the agent. The 72-h time-point was utilized to perform surgery at the orthotopic colon, breast, and head-and-neck cancer models validating the results from the subcutaneous model in clinically more relevant models. At this time point the agent-imaging system combination was able to indicate small tumour nodules on the peritoneum or in the abdominal cavity that were otherwise only detectable by bioluminescence of these cells. Although we did not study the lower boundary of tumour size that could be detected, tumour nodules of 1 mm^3^ could clearly be visualized. Based on the number of cells in spheroids of that size, we estimate the minimal number of cells to be detectable above 50,000. Obviously this detection limit is depending on multiple factors like the imaging system, optical properties of overlying tissue and the probe. Although ROC analysis for the detection of micrometastases showed an excellent sensitivity and specificity at the optimal TBR cut off, it should be noted that the *ex vivo* detection of micrometastases in the peritoneum is very opportune, because almost no background fluorescence is observed in the peritoneum and the detection of metastases is not compromised by any tissue depth. Due to the lack of performance standards for clinically applicable imaging systems as well as imaging agents, at present, the combination of both is of major importance for a successful outcome [44, 45].

Although the anti-EpCAM/800CW conjugate performed well in various tumour types, this probe could still be optimized. The use of antibodies with relatively high affinity, like 323/A3, might culminate in heterogeneous tumour staining, concentrated around the tumour vascularization rather than homogeneous throughout the tumour. The use of an antibody with low or intermediate EpCAM affinity for EpCAM might promote a more homogenous distribution and avoid adverse effects at the cost of a lower total tumour uptake [29, 46]. An improvement in imaging accuracy without compromising the total tumour uptake could be expected by reducing Fc/FcγR interactions through deglycosylation of antibody-based imaging as recently demonstrated by Gao et al. [47]. Further, most EpCAM antibodies target actually the same EGF-like domain of EpCAM. Antibodies identifying other EpCAM domains, like the C- domains might improve detection efficiency [48]. The use of any mouse-derived antibody, like 323/A3, will inevitably induce human anti-mouse antibodies. Although this is not disastrous for imaging purposes, where only one single dose is needed, a chimerized or humanized version with retained efficacy should be preferred [29]. Last but not least, fragments of antibodies like Fab or Fab2 could have higher penetration capabilities compared to full-size antibodies, as shown for several antibodies including anti-EpCAM [35, 38, 49]. Using antibody fragments would lead to shorter incubation times, making clinical translation more applicable. Moreover, recent developments have opened the way for hybrid molecules like immunoenzymosomes, consisting of liposomes equipped with 323/A3 Fab fragments for targeting [50]. Alternative probes for pre- and intraoperative imaging could also be generated by conjugation of the antibody with 2 different labels, one for PET/SPECT and one for NIRF imaging like has been done recently [34, 35, 51, 52].

## Conclusions

FGS is a promising technique to ensure intra-operative fluorescence feedback of tumour margins. Clinical success is only achieved by using a dedicated camera system and tumour-specific agents. This study aimed to achieve complete preclinical validation of an EpCAM targeting fluorescence agent in combination with a next-generation Artemis imaging system. We showed the ability to visualize, primary tumours and millimetre sized tumour nodules and metastases that were otherwise invisible for the human eye. As a novel EpCAM specific optical agent can be used in a wide variety of tumours, together with the knowledge from previous clinical trials and the results from this study, this paves the way for a fast and cost effective clinical translation.

## Abbreviations

BLI: Bioluminescence imaging
EGFR: Epidermal growth factor receptor
EpCAM: Epithelial cell adhesion molecule
FGS: Fluorescence-guided surgery
NIR: Near-infrared
PBS: Phosphate buffered saline
ROC: Receiver operator characteristic
TBR: Tumor-to-background ratio
VEGF(R): Vascular endothelial growth factor (receptor)
